# Mantle Cell Lymphoma of the Palatine Tonsil: A Rare Case Report

**Published:** 2018-09

**Authors:** Ranjitha Rao, Deviprasad Dosemane, Bhagyashree Jaipuria, Debarshi Saha, Manisha Narayan, Kanishka S Rao

**Affiliations:** 1 *Department of Pathology, Kasturba Medical College, Mangalore, Manipal Academy of Higher Education, India.*; 2 *Department of Otorhinolaryngology, Kasturba Medical College, Mangalore, Manipal Academy of Higher Education, India.*

**Keywords:** Immunohistochemistry, Lymphoma, Mantle Cell Lymphoma, Waldeyer’s ring, Tonsil

## Abstract

**Introduction:**

Primary mantle cell lymphoma (MCL) of the palatine tonsil without involvement of the regional lymph nodes is rarely reported.

**Case Report:**

A 52-year-old male presented with complaints of a change in his voice over 3 months, with neither sore throat nor fever. Physical examination revealed right-sided grade IV and left-sided grade III tonsillar enlargement with prominent vessels. The patient underwent bilateral tonsillectomy. An initial histopathological report revealed chronic tonsillitis on the left side and suspicion of atypical lymphoproliferative disorder on the right. Immunohistochemically, the neoplastic cells were positive for Bcl2, CD20, CD5 and Cyclin D1 and negative for CD10, Bcl6 and CD3; thus a diagnosis of MCL was confirmed.

**Conclusion:**

MCL of the tonsil is rare. The microscopic diagnosis is challenging as the picture is very similar compared with other types of small cell lymphomas. A detailed immunohistochemistry panel is required for an accurate diagnosis.

## Introduction

Primary extra-nodal non-Hodgkin lymphomas (NHLs) represent a large proportion of all NHLs, with the most frequent extra-nodal sites being Waldeyer’s ring, the gastrointestinal tract, skin and bones. NHL of Waldeyer’s ring accounts for 5–10% of all primary extra-nodal NHLs. The palatine tonsil is the most frequently (>50%) involved site, followed by the nasopharynx and the base of the tongue (1). Primary mantle cell lymphoma (MCL) of the palatine tonsil without involvement of the regional lymph nodes is rarely reported. Most oral MCLs occur in an elderly male population (2). We report a case of primary MCL of the palatine tonsil in a 52-year-old male patient, with a review of the main features of this subset of extra-nodal lymphoma.

## Case Report

**Fig 1:**
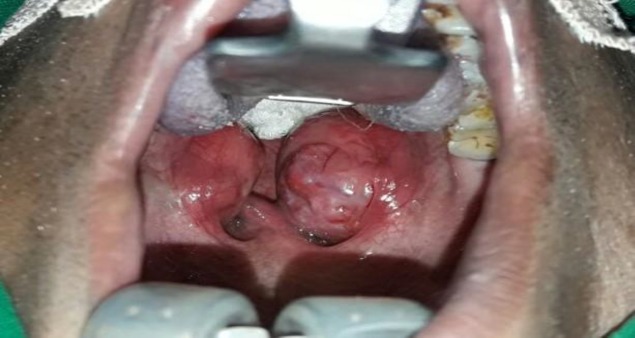
Intraoperative view of the palatine tonsils from the head end of the patient

A 52-year-old male patient presented with a history of a change in the voice over 3 months, with neither sore throat nor fever. Physical examination revealed right-sided grade IV and left-sided grade III tonsillar enlargement with prominent vessels (Fig.1). 

Serology was negative for human immunodeficiency virus (HIV), and other preoperative blood investigations were also normal. He was known to have been diagnosed with type II diabetes mellitus for 15 years, and was treated with oral hypoglycemic medications. Clinical features were not suggestive of chronic tonsillitis, and the possibility of lymphoma was considered due to the asymmetric tonsils and their abnormal surface nodularity and vascularity. Hence, the patient underwent bilateral tonsillectomy by dissection and the snare method. There was no extra tonsillar spread, and no excess bleeding was encountered.

**Fig 2:**
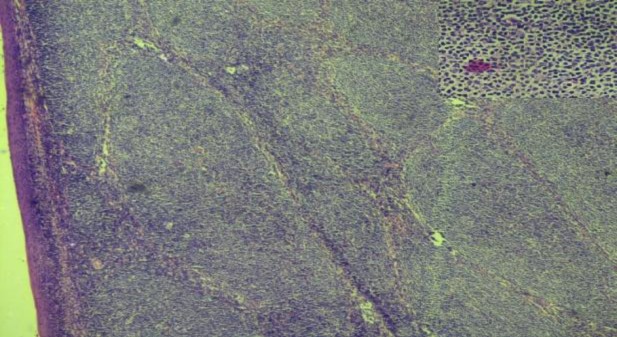
Effaced architecture of the tonsil showing infiltration by the tumor cells arranged in a nodular pattern. The flattened stratified squamous epithelial lining of the tonsil can be seen in the left end of the tissue. 5×, hematoxylin-eosin stain. Inset shows small-to-medium-sized lymphoid cells with irregular nuclear contours, condensed nuclear chromatin, inconspicuous nucleoli and scant cytoplasm. 45×, hematoxylin-eosin stain

On microscopy, the left-side tonsil predominantly showed numerous reactive follicles of various sizes spread throughout the tonsillar tissue. The sections of the right side showed reactive follicles with germinal centers having a darker zone, giving way to a lighter one harboring tingible body macrophages, a polymorphous population of cells including centrocytes, centroblasts and immunoblasts. Some follicles had a broadened mantle cell layer encroaching upon the germinal centers. The mantle layer consisted of a monotonous population of small-to-medium sized lymphoid cells with irregular nuclear contours, condensed nuclear chromatin, inconspicuous nucleoli and scant cytoplasm. A few of the follicles had their germinal centers completely replaced by the mantle-zone cells, imparting a nodular pattern with fairly uniform sizes (Fig. 2). 

**Fig 3:**
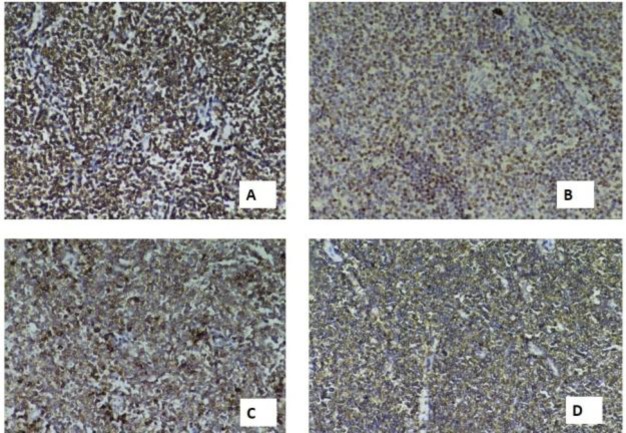
Tumor cells showed positivity for BCL2 (A), Cyclin D1 (B), T-cell associated antigen CD5 (C) and B-cell marker CD20 (D).

Based on these findings, the initial histopathological report suggested chronic tonsillitis with areas suspicious of atypical lymphoproliferative disorder. Immunohisto- chemistry (IHC) was advised for confirmation of the same. With the clinical suspicion of lymphoma already in place, IHC was promptly performed. The neoplastic cells were positive for Bcl2, CD20, CD5 and Cyclin D1. CD10, Bcl6 and CD3 were negative (Fig.3); thus confirming a diagnosis of MCL in both the mantle-zone and nodular patterns. As the patient lived far away from our hospital and wished to take further treatment at an oncology-center close to his home, he was lost to follow-up.

## Discussion

More than half of all NHLs primarily in the head and neck arise in Waldeyer’s ring, while Waldeyer’s ring is the primary site for 5–10% of all NHLs (1). Lymphomas should be included in the differential diagnosis of any patient with a mass in Waldeyer’s ring (3). Tonsillar lymphomas are most commonly of B-cell type, with the most common histological type being the diffuse large B-cell variant (67–96%) and the second most common being MCL (10%). MCL occurs most frequently in men over the age of 60 with a male-to-female ratio of 4:1 (4).

In the study conducted by Salplahta et al, 38 cases of malignant lymphoma were studied, all of which were localized in Waldeyer’s ring. Twenty-one patients presented with unilateral tonsillar swelling, whereas seven cases presented with bilateral tonsillar enlargement. Out of the 38 cases, 29 were diffuse large B-cell lymphoma, making this the most common, whereas two cases (a 61-year-old male and an 81-year-old female) were MCL (5).

Common presenting symptoms are a mass in the throat, dysphagia, odynophagia, sore throat and dysphonia (1). Systemic symptoms are not common until the lymphoma is well-advanced. HIV and Epstein–Barr virus are the most common predisposing factors.

MCL is a lymphoid malignancy of the B-cells of the mantle zone or primary lymphoid follicle. MCL consists of small-to-medium-sized lymphoid cells whose usual differential diagnoses include small lymphocytic lymphoma (SLL), marginal zone lymphoma (MZL) and, occasionally, follicular lymphoma (FL). The typical microscopic appearance of extra-nodal MCL is that of a monomorphic lymphocytic infiltrate that destroys the native architecture, and organizes itself in solid sheets or in vague nodules. Small hyalinized vessels frequently support this malignant infiltrate. Cells are small-to-medium sized and nuclei are oval-to-round with typically inconspicuous nuclei (6). There are three histologic patterns of classic MCL; mantle zone, nodular and diffuse.

The chromosomal translocation t (11;14) (q13; q32), which results in the juxtaposition of the CCND1 (Bcl-1) gene locus to the Ig heavy chain promoter, is a characteristic, but not universal, molecular feature of this lymphoma subtype. This translocation results in the characteristic overexpression of CCND1 protein, a driver of G1/S progression. Immunophenotypic studies have shown that typical cases of MCL express monotypic Ig light chain (more often Ig λ), IgM, IgD, pan-B-cell antigens, BCL-2 and CD5, and are negative for CD3, CD10, CD21, CD23, CD103, MUM1 and BCL-6. Characteristic of MCL is the overexpression of cyclin D1 protein, a feature not seen in other similar-appearing lymphomas (7).

Vasilaki et al reported a case of an 82-year-old male patient with a smooth non-tender mass in the right palatine tonsil. Following bilateral tonsillectomy, immunohistoche- mically the neoplastic cells were positive for CD19, CD20, CD79a, CD22, Bcl 2, CD5 and Cyclin D1 and negative for CD10, CD57, EBV, CD3, CKAE1 and CKAE3. Hence, the diagnosis of MCL of B-phenotype was confirmed (8).

Our patient underwent bilateral tonsillectomy by dissection and the snare method. There was no extra-tonsillar spread and no excess bleeding was encountered. If spread is apparent outside the tonsil, laser or bipolar cautery may be necessary for better clearance of the lesion and hemostasis. Nevertheless, if the tumor is spread laterally to the tonsil, complete removal of the lesion is not necessary as chemotherapy should take care of the remnants. 

Detailed evaluation of the involvement of the lymphatic system elsewhere in the body, such as the gastrointestinal system, is necessary to decide the treatment plan. The gastrointestinal manifestations of MCL include lymphomatous polyposis involving long segments of bowel, usually with mesenteric lymph nodal involvement (1). Treatment varies from combination chemotherapy to chemotherapy and radiotherapy to observation (6). Standard therapy for MCL consists of immunochemo- therapy such as rituximab, cyclophosphamide, hydroxydaunorubicin, vincristine, prednisone (R-CHOP). 

Rituximab-bendamustine followed by maintenance rituximab is increasingly used in the elderly population with MCL. Use of high-dose therapy with autologous stem cell transplantation or dose-intense chemotherapy with fractionated cyclo-phosphamide, vincristine, doxorubicin, dexamethasone (hyper- CVAD) plus rituximab have also shown benefits (3). 

Typically, MCL has a relentless and clinically aggressive course that is resistant to therapy, and a mean survival of only 3-4 years (9). Guggisberg et al reviewed nine cases of MCL in the oral cavity and found the mean survival of patients to be 21 months. However, current results suggest a good prognosis at an early stage (10).

## Conclusion

MCL of the oral cavity is an uncommon diagnosis, most commonly occurring in elderly males. The microscopic diagnosis is challenging as the picture is very similar compared with other types of small cell lymphomas. A detailed immune-histochemical panel is required for an accurate diagnosis. The prognosis for oral cavity MCL appears to be poor, as suggested by literature.
